# A Hypersweet Protein: Removal of The Specific Negative Charge at Asp21 Enhances Thaumatin Sweetness

**DOI:** 10.1038/srep20255

**Published:** 2016-02-03

**Authors:** Tetsuya Masuda, Keisuke Ohta, Naoko Ojiro, Kazuki Murata, Bunzo Mikami, Fumito Tani, Piero Andrea Temussi, Naofumi Kitabatake

**Affiliations:** 1 Laboratory of Food and Environmental Science, Division of Food Science and Biotechnology, Graduate School of Agriculture, Kyoto University, Uji, Kyoto 611-0011, Japan; 2 Division of Applied Life Sciences, Graduate School of Agriculture, Kyoto University, Uji, Kyoto 611-0011, Japan; 3 Department of Basic and Clinical Neurosciences, King’s College, London, London SE59RX (UK); 4 Dipartimento di Chimica, Universita’ di Napoli Federico II, Napoli, I-80126, Italy; 5 Department of Foods and Human Nutrition, Notre Dame Seishin University, Okayama 700-8516, Japan

## Abstract

Thaumatin is an intensely sweet-tasting protein that elicits sweet taste at a concentration of 50 nM, a value 100,000 times larger than that of sucrose on a molar basis. Here we attempted to produce a protein with enhanced sweetness by removing negative charges on the interacting side of thaumatin with the taste receptor. We obtained a D21N mutant which, with a threshold value 31 nM is much sweeter than wild type thaumatin and, together with the Y65R mutant of single chain monellin, one of the two sweetest proteins known so far. The complex model between the T1R2-T1R3 sweet receptor and thaumatin, derived from tethered docking in the framework of the wedge model, confirmed that each of the positively charged residues critical for sweetness is close to a receptor residue of opposite charge to yield optimal electrostatic interaction. Furthermore, the distance between D21 and its possible counterpart D433 (located on the T1R2 protomer of the receptor) is safely large to avoid electrostatic repulsion but, at the same time, amenable to a closer approach if D21 is mutated into the corresponding asparagine. These findings clearly confirm the importance of electrostatic potentials in the interaction of thaumatin with the sweet receptor.

Thaumatin is a sweet-tasting proteins isolated from the fruits of *Thaumatococcus daniellii* Benth, a plant native to tropical West Africa^1^. Thaumatin consists of a single-chain of 207 amino acid residues and is translated as a prepro form with both a 22-amino acid hydrophobic N-terminal extension and an acidic 6-amino acid carboxyl terminal extension^2^,^3^,^4^. On a molar basis, thaumatin is nearly 100,000 times sweeter than sucrose.

We have explored the structure-taste relationship of thaumatin in great depth^5^,^6^,^7^. This knowledge puts us in a privileged position to design new mutants with potentially greater sweetness. The importance of basic residues has long been known for all sweet proteins, as a consequence of the prevalently acidic residues on the surface of the receptor^8^,^9^. Selective chemical modification of thaumatin revealed that five lysine residues (K78, K97, K106, K137, and K187) located on the cleft-containing side are essential for sweetness^5^. Site-directed mutagenesis of the remaining 4 lysine residues on this side (K19, K49, K67, and K163) confirmed that most lysine residues on this surface are involved in eliciting sweetness, and particularly K67 is important^6^,^7^. On the contrary, the possible effects of acidic amino acid residues on the structure-activity relationship of thaumatin are not fully understood.

Herein, to clarify the effects of acidic amino acid residues on the sweetness of thaumatin and to design sweeter forms of the protein, we report site-directed mutagenesis of crucial negatively charged residues. The structure of thaumatin hosts 19 acidic residues, but it is possible to restrict the choice using, as a guide, previous knowledge on the most likely region of the protein surface that interacts with the receptor^5^ and preliminary results of docking studies, based on the so-called wedge model for the interaction of sweet proteins with the sweet taste receptor^9^. Current knowledge is that a single receptor accounts for the sweet taste of all sweet molecules, from sugars to sweet proteins: it is a metabotropic G-protein-coupled receptor (GPCR) composed of two similar peptide chains, T1R2 and T1R3^10^,^11^. Typically, a metabotropic GPCR is formed by three domains: a Venus flytrap domain (VFTD), a cysteine rich domain (CRD), and a seven helices transmembrane domain (TMD). Although it was immediately accepted that most small sweet molecules bind to the cavities of the VFTD, it proved more difficult to account for the interaction of sweet proteins, mainly because of their dimensions. Considering that the molecular volumes of thaumatin and aspartame can be estimated as 27,000 Å^3^ and 270 Å^3^ respectively, it is difficult to imagine that sweet proteins can bind to the same sites of the sweet receptor that bind small ligands. The first interpretation of the mechanism of interaction of sweet proteins with the sweet receptor was proposed by Temussi^12^ soon after the discovery of the sweet receptor. This mechanism is known as “wedge model”. As large molecules cannot be hosted by the orthosteric binding sites of the VFTD of either T1R2 or T1R3 protomers, several hypotheses were formulated. Cell-based assays suggested that the amino-terminal domain of T1R2 is required for responses to monellin, brazzein, aspartame, and neotame^13^,^14^, the CRD of human T1R3 is essential for response to brazzein and thaumatin^14^,^15^, and the amino-terminal domain of T1R3 is required for neoculin^16^. Five amino acid residues in the CRD of T1R3 are important for the response to thaumatin, but as the dimension of a typical CRD is not consistent with the large surface spun by key residues of thaumatin^17^ an alternative explanation may be formulated. It is likely that even a slight tilt of the VFTD towards the membrane surface, induced by tampering with the architecture of the CRD, would prevent binding as foreseen in the wedge model. Altogether, no direct observations of the interaction of sweet proteins with the receptor have been put forward. Accordingly, the wedge model remains the only viable working model to interpret the structure-activity relationship of sweet proteins.

The basis of the wedge model is the homology between the sweet receptor and the mGluR1 glutamate receptor, the template used to build the homology model of T1R2-T1R3^18^. It was postulated that the sweet receptor, like mGluR1, can exist as an equilibrium mixture between a resting conformation and the active form, even when the ligands are not bound. Small molecular weight sweet compounds shift the equilibrium by entering one or two of the orthosteric sites inside the VFT domains. Larger molecules like sweet proteins achieve the same result by binding to a secondary, external site of the active form. In the case of thaumatin the region interacting with the receptor is approximately coincident with the so-called cleft-containing face of the protein^5^. The two most important basic residues, namely K67 and R82, are at the center of this region and are surrounded by six acidic residues: D21, E42, D55, D59, D60, and E89. We prepared six mutant thaumatin constructs (D21N, E42Q, D55N, D59A, D60A, and E89Q) and had their sweetness evaluated by a human panel. The results show that most of the acidic residues do not play a significant role in eliciting sweetness. However, removal of the negatively charged residue D21 produced the sweetest thaumatin mutant, one of the two sweetest known proteins. Models derived from tethered docking provide a deeper insight in our understanding of how thaumatin and its D21N mutant interact with the sweet receptor.

## Results

### Design of thaumatin mutants

The effects of charged residues on the elicitation of sweetness have been thoroughly investigated in other sweet-tasting proteins, such as brazzein, monellin, neoculin (curculin), and lysozyme^8^,^13^,^19^,^20^,^21^,^22^,^23^,^24^,^25^ suggesting that sweet-tasting proteins interact with sweet receptors through a multi-point interaction involving both positive and negative charges but mainly basic residues. Thus, mutations at acidic residues are expected to produce more potent sweeteners only when they increase the basicity of the proteins. However, this view is highly reductive, implying that the surface of interaction is uniformly charged. In reality the alternation of basic, acidic and neutral residues in an entangled patchwork is ultimately responsible of the extremely high affinity of sweet proteins to the sweet receptor. During the past few years we have explored the structure-activity relationship of thaumatin in great depth^5^,^6^,^7^,^26^,^27^,^28^,^29^,^30^. We capitalized on such knowledge to choose the right acidic residues to mutate. As mentioned in the introduction sweet proteins can activate the receptor by binding to an external site of the active form whose surface is largely acidic. In the case of thaumatin the binding region, roughly coincident with the so-called cleft-containing face of the protein^27^, hosts, at its center, two crucial basic residues, K67 and R82, surrounded by six acidic residues: D21, E42, D55, D59, D60, and E89. We focused on these acidic residues to design thaumatin mutants.

**Figure 1:**
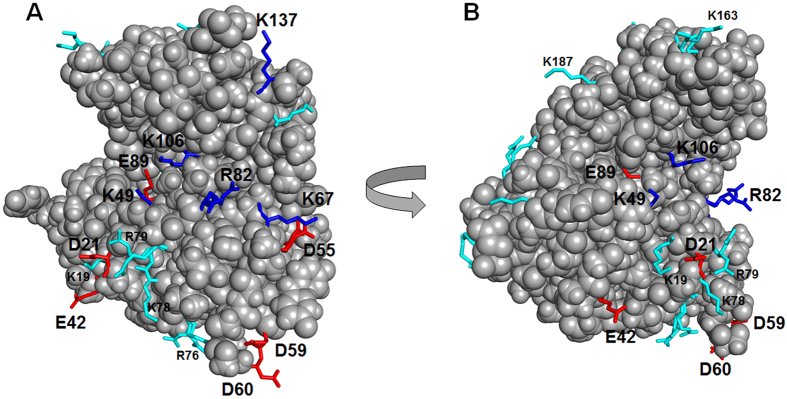
Overall view of thaumatin. Two views of thaumatin showing the relative positions of key basic residues and acidic residues chosen for mutagenesis. All the acidic residues investigated (D21, E42, D55, D59, D60, and E89) are shown in red, whereas basic ones are either in cyan (when far below the plane of panel (**A**), or blue when close to the surface of interaction. Panels (**A**,**B**) are related by a 90° rotation. Molecular models were generated with *PyMOL*^50^.

At first, we investigated the effects of D55, which is in the vicinity of one of the critical residues, K67. Upon removal of a carboxyl group, the positively charged patch around K67 would be widened and the sweetness of the mutant might be affected. However, no significant effect on sweetness was observed. Although the molecular surface of thaumatin is relatively positively charged, two neighboring aspartic acid residues, D59 and D60, form acidic patches (Fig. 1). However, the results indicated that D60 is not important for sweetness. This finding well coincides with a previous chemical modification study, suggesting that D60 did not play a significant role in the sweetness of thaumatin^5^. Mutation of D59 resulted in a slight increase in the threshold value (87 nM). Taken together, the thaumatin binding site to the sweet receptor rather than the side hosting the cleft region appears to be centred around the ridge of the three basic amino acid residues K67, R79, and R82^6^. Next, we examined the effects of D21 and E42, which are also located in the vicinity of the ridge (Fig. 1). Although a previous study suggested that modification with pyridoxamine at D21/E42/D129 resulted in a slightly sweeter thaumatin^5^, the contribution of each residue has not been fully proven. Mutation E42Q had no significant effect on sweetness, suggesting E42 to be outside of the receptor-binding site. The structure of thaumatin is homologous to those of non-sweet thaumatin-like protein, such as PR-5, zeamatin, and osmotin^31^,^32^,^33^. Most of the differences among them are in the thaumatin loop which is composed of 7 amino acid residues (D-A-A-L-D-A-G). D21 is located in this thaumatin loop, which is not found in other non-sweet thaumatin-like proteins. Interestingly, the mutation D21N resulted in increased sweetness. Altogether we designed and expressed the following mutant thaumatin constructs: D21N, E42Q, D55N, D59A, D60A, and E89Q. The rationale for this choice can be drawn from Fig. 1 where it can be seen that most acids are too far away (E42, D59, and D60) or shielded (E89 by K106 and D55 by K67). The only remaining residue is D21 which, upon a small rearrangement of the protein orientation, can come close to the surface (*vide infra*).

### Structural characterization of the thaumatin mutants

**Figure 2:**
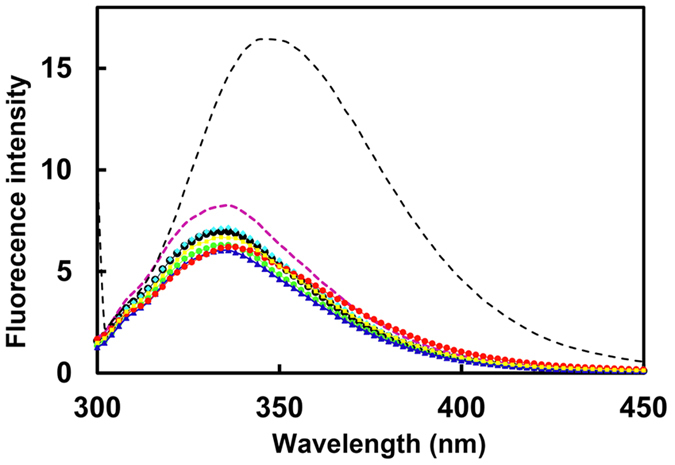
Fluorescence spectra of mutant thaumatin proteins. Plant thaumatin (black circle), D21N (red circle), E42Q (purple dot), D55N (blue triangle), D59A (green diamond), D60A (cyan diamond), E89Q (yellow square). Plant thaumatin denatured by 6 M urea (black dot) were excited at 280 nm and emission spectra were recorded at 25 °C.

**Figure 3:**
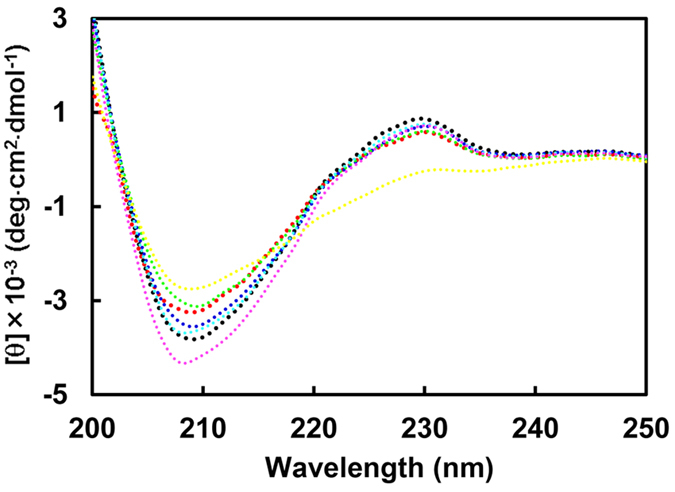
Far-UV CD spectra of mutant thaumatin proteins. CD spectra were recorded in 5 mM sodium phosphate buffer, pH 7.0, as described in Experimental procedures. Plant thaumatin (black dot), D21N (red dot), E42Q (purple dot), D55N (blue dot), D59A (green dot), D60A (cyan dot), E89Q (yellow dot).

The six amino acid residues mutated in the present study are indicated in red in Fig. 1, alongside representative basic residues previously suggested as key glucophores. The purity of the thaumatin mutants obtained after purification was confirmed by SDS-PAGE and native PAGE (Supplementary Figure S1). A single band with a molecular size similar to that of plant thaumatin was obtained in SDS-PAGE, suggesting that the mutant proteins are of high purity (Supplementary Figure S1A). Native PAGE showed that the mobility of the band increased when D or E were substituted with N, Q, or A (Supplementary Figure S1B), because a charge-specific mutation would be introduced into the thaumatin molecule. To clarify whether mutations induced relevant structural changes fluorescence and CD spectra analyses were performed. No significant peak shift and no change in fluorescence intensity of mutants was observed compared to that of plant thaumatin I in the fluorescence spectra (Fig. 2). These results suggest that no gross conformational change around Trp residues was induced by the mutations. In CD spectra, all the mutants, except E89Q, have profiles similar to that of plant thaumatin, suggesting that no obvious change in secondary structure was induced by the mutations, with the exception of E89Q (Fig. 3). Estimation of protein secondary structure content from each CD spectra was performed by means of software K2D3^34^. The results (Supplementary Table S1) suggest that no substantial conformational changes are induced by the mutations, including E89Q.

### Sweetness of the thaumatin mutants

The results of the sensory analysis by triangle-test are shown in Table 1. The threshold values of all mutants except D21N ranged from 57 nM to 87 nM, and a remarkable difference was not observed compared to plant thaumatin. Since E42Q, D55N, D59A, and D60A have a structure similar to that of plant thaumatin, it is fair to say that negatively charged residues such as E42, D55, D59, and D60 on the cleft-containing side are not critically involved in thaumatin’s sweetness elicitation. As to E89, the structure around strand F is not required for sweetness, and these areas would not interact with the sweet receptor. It is significant that, as shown by tethered docking (*vide infra*) E89 cannot interact with the receptor because its side chain is completely shielded from the surface by K106. The same is true for D55, shielded by K67. Mutation D21N resulted in increased sweetness without any significant conformational change in the molecule as assessed using tryptophan fluorescence and CD spectra. The mutant has a threshold value of 31 nM, making it approximately 150,000-fold sweeter than sucrose on a molar basis and the sweetest protein identified. D21 is located in a thaumatin loop not found in non-sweet thaumatin-like proteins^31^,^32^,^33^.

### How does thaumatin interact with the sweet receptor?

**Figure 4:**
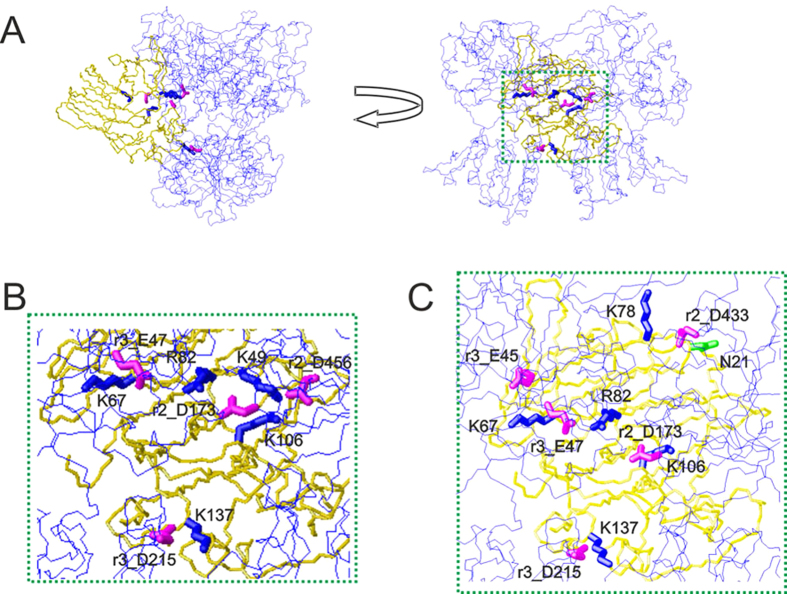
The wedge complex of thaumatin and D21N thaumatin with the T1R2-T1R3 receptor. (**A**) Two views of the complex related by a 90° rotation. The model of the receptor is shown as a line representation (blue) of the backbone whereas the model of thaumatin is shown as a neon representation of the backbone (gold). The side chains of the key basic residues of thaumatin chosen to optimize the complex are represented as thick blue neons. The corresponding side chains of the acidic residues of the receptor are represented as magenta neons. (**B**) Enlargement of the interaction zone surrounded by green dots in panel (**A**). This view shows the side of the sweet protein in contact with the receptor. Receptor residues are labeled with the prefix r2 when belonging to the T1R2 protomer and with r3 when belonging to the T1R3 protomer respectively. (**C**) Corresponding enlargement of the complex of D21N thaumatin with the T1R2-T1R3 receptor. The area of contact with the receptor is larger than that of wild type thaumatin. Models were built with MOLMOL^51^.

**Figure 5:**
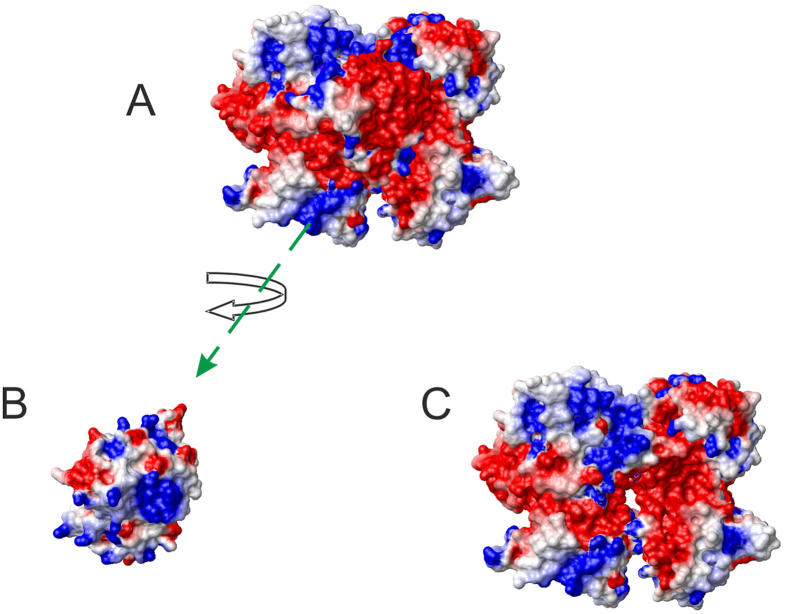
Electrostatic surface of the wedge complex of D21N thaumatin with the T1R2-T1R3 receptor. (**A**) Molecular model of the whole complex. (**B**) Molecular model of D21N thaumatin rotated and displaced to show the inner surface. (**C**) Molecular model of the T1R2-T1R3 receptor alone showing the interacting surface. Models were built with MOLMOL^51^.

In order to explain how thaumatin interacts with the receptor and particularly to interpret the puzzling case of the D21N thaumatin mutant we resorted to the first mechanism proposed to interpret the interaction of sweet proteins and their receptor, the so-called wedge model^12^,^35^. In formulating this model, we used a specific *in silico* docking procedure, GRAMM^36^, because it can be used at low resolution^12^,^35^. This choice was instrumental to validate the wedge model but yields only fuzzy representations of potential complexes. More recently, it was possible to build more accurate topological models of the receptor with two sweet proteins, brazzein and monellin, by applying a tethered docking procedure in which the use of mutagenesis data and the distribution of charged residue on the interface between protein and receptor plays a major role^9^. Thus, we attempted to build accurate models for thaumatin by using this procedure resorting to mutagenesis data for choosing the most reliable orientation among the complexes originally furnished by GRAMM^12^. Subsequently, the models were refined by imposing the consistency of all mutual motions with the mutagenesis data as mentioned previously^9^. The best molecular model of the complex yielded by tethered docking is shown in Fig. 4. It has been observed that the surface of interaction of the T1R2-T1R3 receptor with sweet proteins, although very complex, is prevalently negative^12^,^35^. We have been able to support this view by assessing the effect of mutations of basic residues on the surface of thaumatin. We have shown that four lysine residues, but mainly K67, and three arginine residues, mainly R82, are critical in determining thaumatin sweetness^6^,^7^. It was thus rather surprising to find that the D21N mutation is not adverse or simply neutral with respect to the sweetening power of thaumatin but increases it considerably. Therefore we set to investigate this finding in the framework of existing models of interaction of sweet proteins with their receptor. The most important interface residues found with this procedure were K49, K67, K106, K137, and R82, and each of them is close to a receptor residue of opposite charge. The main contacts between receptor residues and thaumatin residues are as follows. The CZ atom of R82 of thaumatin is at 4.97 Å from the CG atom of D173 of the receptor; likewise NZ of K67 is 5.36 Å from CB of E47 of T1R3, NZ of K137 is 5.36 Å from CB of D215 of T1R3, NZ of K106 is 4.96 Å from CG of D173 of T1R2 and NZ of K49 is 6.02 Å from the CG atom of D456 of T1R2. All distances are small enough that proper conformational changes of the side chains can yield optimal electrostatic interaction. The closest distance of D21 from acidic residues of the receptor is 12.1 Å from its CG atom to the CG atom of D433 (CG) of T1R2. It is a comfortable distance to avoid electrostatic repulsion but, at the same time, amenable to a closer approach if D21 is mutated into the corresponding asparagine. Applying the same approach to D21N thaumatin we got a similar but not identical result. The distance between CG of N21 to CG of D433 of T1R2 drops to 6.10 Å and, even more significantly, the interaction between K67 and E47 of T1R3 is flanked by a new interaction of K67 with E45 of T1R3. K78 also comes into prominence as an important player. The molecular model of the complex between receptor T1R2-T1R3 and D21N thaumatin is shown in Fig. 4C. The main contacts between D21N thaumatin residues and receptor residues can be summarized as follows. The CZ atom of R82 of thaumatin is at 4.63 Å from the CG atom of D173 of T1R2; likewise NZ of K67 is 6.66 Å from CB of E47 of T1R3 and 6.27 Å from CG of E45 of T1R3, NZ of K137 is 4.98 Å from CB of D215 of T1R3, NZ of K106 is 4.98 Å from CG of D173 of T1R2 and NZ of K78 is 4.51 Å from CG of D433 of T1R2. All these tight interactions reflect a good complementarity of the electrostatic surfaces of interaction (Fig. 5). The electrostatic surface of the whole complex is shown in Fig. 5A, whereas the two interacting surfaces are shown in Fig. 5B, C for D21N thaumatin and the T1R2-T1R3 receptor respectively.

## Discussion

Life-style related diseases such as hypertension, hyperlipidemia, diabetes, and obesity have become major problems in the world. These diseases seem to be linked to staggering increases in obesity. However, as sweetness is an important taste, the use of the low-calorie sucrose substitutes in foods, beverages, and medicines should be considered. Sweet-tasting proteins are potential low-calorie substitutes for sugars. Thaumatin elicits a sweet taste at a low concentration, approximately 100,000-fold less than that of sucrose on a molar basis. In the hope of using more potent sweeteners the biotechnological production of sweet-tasting proteins has been implemented^37^,^38^ but to design proteins even sweeter than natural ones it is essential to clarify the mechanisms for the elicitation of sweetness. Understanding the mechanism of action of sweet proteins has been a challenging problem from the start, soon after the discovery of taste receptors^10^, essentially for two reasons: there is a single receptor for all sweet molecules^11^ and there are no commonalities among sweet proteins^39^. The single sweet taste receptor is a heterodimer of a class C GPCR made of two slightly different protomers^40^,^41^,^42^,^43^,^44^,^45^, each hosting three domains: a Venus flytrap domain (VFTD), a cysteine rich domain (CRD), and a seven helices transmembrane domain (TMD). As mentioned in the introduction, large molecules cannot be hosted by the orthosteric binding sites of the VFT domains of either T1R2 or T1R3 protomers. This circumstance among other considerations led us to focus on the wedge model^12^,^39^ to interpret the puzzling case of the D21N thaumatin mutant. Recently, we have shown that it is possible to build accurate topological models of the complexes of the receptor with a sweet protein by applying a tethered docking procedure in which the distribution of charged residue on the interface between protein and receptor plays a major role^9^. Application of this procedure to thaumatin is facilitated by the availability of high quality mutagenesis data concerning key charged residues^6^,^7^. Here we applied tethered docking to sort out the best complex of T1R2-T1R3 with thaumatin among the many yielded by GRAMM^35^ and then check its consistency with the surprising D21N mutation. The best molecular model of the complex between thaumatin and receptor suggested that each of the charged residues of thaumatin previously identified as crucial^5^,^6^,^7^ is close to a receptor residue of opposite charge. Interestingly, the model between D21N thaumatin and the receptor yielded a similar but not identical result. The distance between CG of N21 to CG of D433 significantly drops, and the interaction between K67 and E47 of T1R3 is flanked by a new interaction of K67 with E45 of T1R3. K78 also came into prominence as an important player. These subtle rearrangements would lead to an enlarged area of interaction and thus presumably enhance the sweet taste of thaumatin. Altogether, the two complexes found for wild type thaumatin and D21N thaumatin solve a rather mysterious case. In line with the prevalently negative electrostatic potential of the receptor, it has been shown before that mutations changing acidic or neutral residues into basic ones can enhance the sweetness of proteins. In fact Y65R mutation generated a hypersweet monellin^8^. That simply making an acidic residual neutral could produce a hypersweet thaumatin was rather surprising. The possibility of rationalizing not only the interaction with the receptor of wild type thaumatin but also to explain the puzzling increase of sweetness brought about by the D21N mutation lends strong support to the wedge model as a reliable mechanism of interaction of all sweet proteins with the sweet taste receptor. The sweetest mutant D21N thaumatin could provide a good clue for designing more effective sweeteners and would help our understanding of the mechanism of interaction of thaumatin with sweet receptors.

## Methods

### Materials

Plant thaumatin was purified from crude thaumatin powder by ion exchange column chromatography followed by gel filtration as described previously^5^.

### Site-directed mutagenesis

The vector pCR2.1-PreTH, carrying both the pre sequence and a mature thaumatin gene, was used as the template for mutagenesis^4^. Site-directed mutagenesis was performed using a QuikChange kit (Stratagene, La Jolla, CA, USA) with two oligonucleotide primers complementary to opposite strands and containing the desired mutation (Supplementary Table S2). The mutations were confirmed using an ABI 310 DNA sequencer (Applied Biosystems, Warrington, UK). The plasmid containing the desired mutation was digested by *Csp*45 I and *Xba* I, and ligated with the yeast shuttle vector pPIC6*α* previously digested with the same restriction enzymes. The mutated plasmid was digested by *Pme* I and introduced into *Pichia* X-33 by electroporation as described previously^46^,^47^.

### Purification of thaumatin mutants

Expression and secretion of the thaumatin mutants from the yeast *Pichia pastoris* were performed by 2 L or 7 L of fermentor as described previously^6^,^46^,^47^. Average protein yields ranged approximately from 50 to 100 mg/L. Purification of the thaumatin mutants was performed by cation exchange chromatography as described previously^6^,^47^. In brief, the culture supernatant was dialyzed against 5 mM sodium phosphate buffer, pH 7.0, containing 0.5 mM EDTA at 4 °C for 2 days. Subsequently, the dialysate was applied to an SP-Sephadex C-25 (Amersham Bioscience) column previously equilibrated with 5 mM sodium phosphate buffer, pH 7.0, and eluted with 5 mM sodium phosphate buffer, pH 7.0, containing 0.5 M NaCl. The fractions containing proteins were collected, and precipitated by ammonium sulfate (final. 75%). After centrifugation at 8,000 g for 20 min, the precipitate was dissolved in 5 mM sodium phosphate buffer, pH 7.0 and then dialyzed against 5 mM sodium phosphate buffer, pH 7.0. The dialysate was applied to a Toyopearl HW-50F column (Tosoh, Tokyo, Japan) and eluted with a hepes buffer, pH 7.0 containing up to 0.15 M NaCl. Protein purity was examined by SDS-PAGE and native PAGE as described below. The concentration of mutant thaumatin proteins was determined by a BCA analysis using thaumatin I purified from crude thaumatin powder as a standard.

### Polyacrylamide gel electrophoresis (PAGE)

SDS-PAGE was performed in a 13.5% gel according to the method of Laemmli^48^. Native PAGE was performed using a 10% homogeneous native polyacrylamide gel for the basic protein^49^. The gels were stained with Coomassie Brilliant Blue R-250.

### Sensory analysis

The sweetness threshold of the samples was evaluated by a triangle test for taste threshold^5^,^25^. Three paper cups, one containing 5 mL of protein solution and the others containing 5 mL of distilled water, were prepared. Sweetness intensity was evaluated on a scale of 0 to 5 using a scaling bar. The value 0 means no taste detected from the test solution; 1 means that the sample solution elicited some taste stimulation; and 2 represents the concentration at which the panel member detected sweetness from the sample solution. That is, the threshold value of sweetness is the concentration giving a value of 2. First sensory test was performed using the following concentrations of 10, 20, 50, 70, 100, and 200 nM for all mutants. The second sensory analysis was performed using the following the concentrations of 5, 10, 20, 30, 40, and 50 nM for D21N, and 10, 20, 30, 50, and 80 nM for D55N as well as plant thaumatin. The threshold values were averaged and analyzed with one-way analysis of variance. A post hoc test was performed by Bonferroni and Scheffe test. A *P* < 0.05 value was considered as a significant difference in the statistical analysis.

### Fluorescence spectra

Fluorescence spectra of mutant thaumatin proteins were recorded in 5 mM sodium phosphate buffer, pH 7.0, at 25 °C on a fluorescence spectrophotometer (F-3000; Hitachi, Ltd., Tokyo, Japan) with an excitation wavelength of 280 nm; the emission wavelength ranged from 300 to 450 nm. The excitation and emission band passes were set at 3 nm. The protein concentration was adjusted to 5.7 *μ*M.

### Circular dichroism (CD) spectra

CD spectra were recorded with a J-720 spectropolarimeter (Jasco, Tokyo, Japan). The protein concentration was 22.5 *μ*M in a 1 mm cell at wavelengths from 250 to 200 nm. Spectra for one sample were recorded three times and mean residue ellipticities are given. Estimation of protein secondary structure from each CD spectra was performed by using K2D3^34^.

### Representation of the thaumatin molecule

The molecular model of thaumatin was prepared with the *PyMOL* program^50^ or MOLMOL^51^ using PDB data for thaumatin I (PDB entry: 3AL7^27^).

### Homology modelling

ClustalX^52^ was employed to get sequence alignments. The molecular model of D21N thaumatin was built using SWISS MODEL and the high resolution X-ray structure of thaumatin as template (PDB entry: 3AL7). All T1R2-T1R3 models were built using SWISS MODEL (EXPASY) in the oligomeric mode^36^ using the relevant crystal structures of mGluR1 as templates^18^. In particular, we used PDB entry 1EWT for free form I, and PDB entry 1EWK for free form II^18^.

### Protein docking

As previously described^9^, ensembles of complexes of thaumatin (PDB entry: 3AL7) with the T1R2-T1R3 receptor models were obtained from the GRAMM software in its low resolution mode^36^. All models were visualized using MOLMOL^51^. Refinement of the thaumatin-receptor complexes was done using GRAMM-X, the web version of GRAMM (http://vakser.bioinformatics.ku.edu/resources/gramm/grammx). Using this modality it is possible to add virtual receptor interface residues taken from the low resolution complexes, receptor residues required, interface residues taken from mutagenesis data, and the minimum number of pairs of receptor-ligand residues to be retained in the refined complex. Among receptor residues, we favored charged residues, both because of the importance of electrostatic interactions in the wedge model and also because two member interactions are easier to handle than those among several apolar residues, as for instance hydrophobic interactions. The following residues were selected: (T1R2) D169, E170, R172, D173, K174, R176, D213, R217, D218, D456, R457, and (T1R3) R177, D190, R191, D216. In the case of thaumatin we chose K49, K67, K78, K97, K106, K137, K163, K187, R76, R79, and R82 for preferred ligand interface residues. The other parameters were maximized as described before^9^.

## Additional Information

**How to cite this article**: Masuda, T. *et al.* A Hypersweet Protein: Removal of The Specific Negative Charge at Asp21 Enhances Thaumatin Sweetness. *Sci. Rep.* **6**, 20255; doi: 10.1038/srep20255 (2016).

## Supplementary Material

Supplementary Information

## Figures and Tables

**Table 1:** Sensory analysis of mutant thaumatin proteins.

	Sweetness threshold (nM)
Plant §	51 ± 4
D21N §	31 ± 4*
D55N §	57 ± 4
E42Q ¶	65 ± 13
D59A ¶	87 ± 13**
D60A ¶	63 ± 7
E89Q ¶	68 ± 2

The sweetness of mutant thaumatin proteins is represented as the threshold value (mean ± SE). § n = 7, ¶ n = 3. **Significance among all mutants’ data (*P* < 0.05), *Significance among Plant, D21N, and D55N data (*P* < 0.01).
