# Supplementary material for: A Hypersweet Protein: Removal of The Specific Negative Charge at Asp21 Enhances Thaumatin Sweetness
Source: Sci Rep. 2016 Feb 3;6:20255. doi: 10.1038/srep20255 (PMC4738316; doi:10.1038/srep20255)
Supplement: Supplementary Information [file srep20255-s1.pdf]

Title: **A Hypersweet Protein: Removal of The Specific Negative Charge at Asp21 Enhances Thaumatin Sweetness**

Author: **Tetsuya Masuda[1]*, Keisuke Ohta[1], Naoko Ojiro[1], Kazuki Murata[1], Bunzo Mikami[2], Fumito Tani[1], Piero A. Temussi[3,4]* and Naofumi Kitabatake[5]**

Affiliation: [1]Laboratory of Food and Environmental Science,
Division of Food Science and Biotechnology,
Graduate School of Agriculture, Kyoto University,
Uji, Kyoto 611-0011, Japan
[2]Division of Applied Life Sciences,
Graduate School of Agriculture, Kyoto University,
Gokasho, Uji, Kyoto, 611-0011, Japan
[3] Department of Basic and Clinical Neurosciences, King’s College London, London SE59RX (UK)
[4]Dipartimento di Chimica, Universita' di Napoli Federico II, Napoli, Italy.
[5]Department of Foods and Human Nutrition,
Notre Dame Seishin University, Okayama 700-8516 Japan

***Corresponding author:**

Tetsuya Masuda

Division of Food Science and Biotechnology,

Graduate School of Agriculture,

Kyoto University,

Uji, Kyoto, Japan.

Tel: +81-774-38-3741, Fax: +81-774-38-3740.

E-mail: t2masuda@kais.kyoto-u.ac.jp

Piero A. Temussi

Dipartimento di Scienze Chimiche, via Cinthia 45, Naples 80126,

Universita' di Napoli Federico II,

Napoli, Italy.

Tel +39 081674416, fax: +39 081674409.

E-mail: temussi@unina.it

Supplementary Table S1

Estimation of secondary structure from CD spectra

	α-helix	β-strand
Plant	1.04	43.34
D21N	1.09	43.26
E42Q	1.30	43.03
D55N	1.10	43.55
D59A	0.84	43.97
D60A	1.04	43.48
E89Q	1.09	43.86

Estimation of secondary structure contents of α-helix and β-strand (%) was performed by K2D3 web version (http://cbdm-01.zdv.uni-mainz.de/~andrade/k2d3//).

Supplementary Table S2 Primers for site-directed mutagenesis

**D21N**		
	**sense**	**GCCTCCAAAGGCAACGCCGCCTGGAC**
	**antisense**	**GTCCAGGGCGGCGTTGCCTTTGGAGGC**
**E42Q**		
	**sense**	**GACCATCAACGTACAACCCGGCACCAACG**
	**antisense**	**CGTTGGTGCCGGGTTGTACGTTGATGGTC**
**D55N**		
	**sense**	**GCCCGCACCAACTGCTATTTCGACGACAGC**
	**antisense**	**GCTGTCGTCGAAATAGCAGTTGGTGCGGGC**
**D59A**		
	**sense**	**CCGACTGCTATTTCGCCGACAGCGGCAGC**
	**antisense**	**GCTGCCGCTGTCGGCGAAATAGCAGTCGG**
**D60A**		
	**sense**	**GACTGCTATTTCGACGCCAGCGGCAGCGG**
	**antisense**	**CCGCTGCCGCTGGCGTCGAAATAGCAGTC**
**E89Q**		
	**sense**	**CCACCACGCTGGCGCAGTTCTCGCTCAACC**
	**antisense**	**GGTTGAGCGAGAACTGCGCCAGCGTGGTGG**

A Hypersweet Protein:

Removal of The Specific Negative Charge at Asp21 Enhances Thaumatin Sweetness

Tetsuya Masuda et al.

Supplementary Figure S1

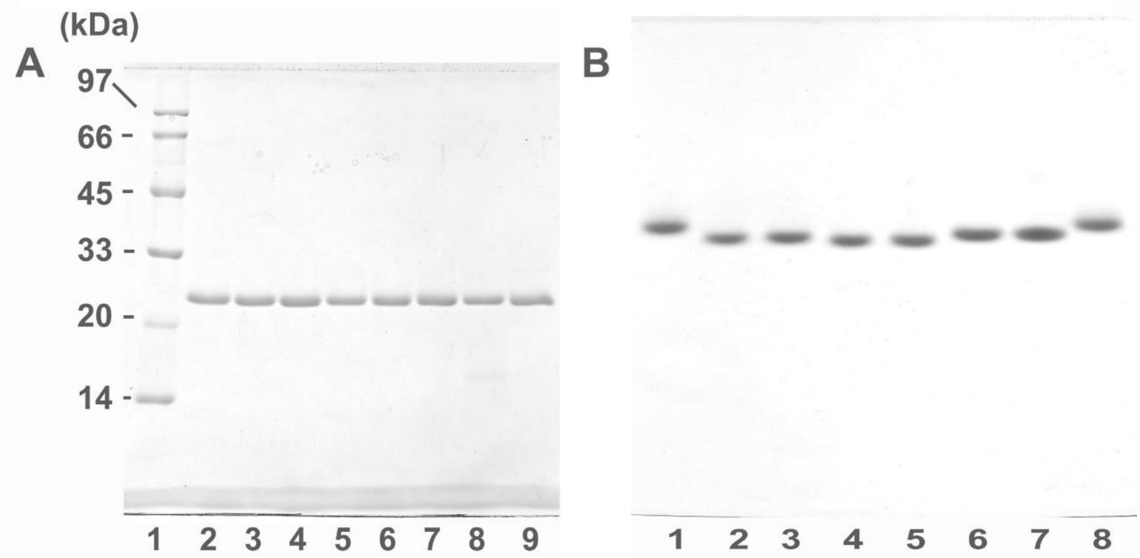

Supplementary Figure S1. SDS-PAGE and native-PAGE of mutant thaumatin proteins. (A) SDS-PAGE on a 13.5% gel and staining with Coomassie Brilliant Blue. Lane 1, M.W. marker; lane 2, 9, plant thaumatin; lane 3, D21N; lane 4, E42Q; lane 5, D55N; lane 6, D59A; lane 7, D60A; lane 8, E89Q. (B) Native-PAGE on a 10.0% gel and staining with Coomassie Brilliant Blue. Lane 1, 8, plant thaumatin; lane 2, D21N; lane 3, E42Q; lane 4, D55N; lane 5, D59A; lane 6, D60A; lane 7, E89Q.

A Hypersweet Protein:

Removal of The Specific Negative Charge at Asp21 Enhances Thaumatin Sweetness

Tetsuya Masuda et al.
